# Primary End‐to‐End Repair of Subacute Extensor Hallucis Longus Tendon Rupture Supported by a Preserved Extensor Hallucis Capsularis: A Case Report

**DOI:** 10.1155/cro/4752831

**Published:** 2026-04-15

**Authors:** Ryo Inoue, Yuki Suzuki, Masanari Hamasaki, Yukinori Tsukuda, Shigeto Hiratsuka, Katsuro Ura, Takuya Ogawa, Masatake Matsuoka, Koji Iwasaki, Tomohiro Onodera, Eiji Kondo, Norimasa Iwasaki

**Affiliations:** ^1^ Department of Orthopaedic Surgery, Otaru General Hospital, Otaru, Hokkaido, Japan; ^2^ Department of Orthopaedic Surgery, Faculty of Medicine and Graduate School of Medicine, Hokkaido University, Sapporo, Hokkaido, Japan, hokudai.ac.jp; ^3^ Department of Functional Reconstruction for the Knee Joint, Faculty of Medicine, Hokkaido University, Sapporo, Hokkaido, Japan, hokudai.ac.jp; ^4^ Centre for Sports Medicine, Hokkaido University Hospital, Sapporo, Hokkaido, Japan, hokudai.ac.jp

**Keywords:** case report, extensor hallucis capsularis, extensor hallucis longus, suture, tendon rupture

## Abstract

Extensor hallucis longus (EHL) tendon rupture is an uncommon injury that usually results from trauma and often requires surgical intervention. In the subacute phase, primary tendon repair becomes challenging because of tendon retraction, and tendon transfer or graft reconstruction is often necessary. Anatomical variations of the EHL, particularly the extensor hallucis capsularis (EHC), may influence tendon retraction and reparability, although their clinical significance has rarely been reported. We present a rare case of subacute EHL rupture in which primary end‐to‐end repair was feasible owing to the presence of a preserved EHC. The purpose of this case report is to highlight the surgical and anatomical significance of the EHC, specifically the Olewnik Type IIb variant, in preserving tendon alignment and enabling delayed primary repair. A 26‐year‐old woman presented with loss of hallux extension 1 week after a sharp laceration. Magnetic resonance imaging revealed complete EHL rupture. During surgery performed 11 days postinjury, the EHC was identified as a medial accessory tendon inserting distal to the extensor hallucis brevis insertion, consistent with the Olewnik Type IIb classification. The EHC appeared to prevent proximal tendon migration, leaving a 5‐mm tendon gap and enabling a six‐strand Yoshizu Type I repair. Rehabilitation began at 2 weeks, and by 12 weeks the patient achieved full strength and motion without complications. This case demonstrates that preservation of the EHC can maintain partial extensor mechanism continuity and may expand the indications for primary repair in carefully selected subacute cases. Awareness of this anatomical variant may assist in preoperative planning and surgical decision‐making.

## 1. Introduction

Extensor hallucis longus (EHL) tendon rupture is a relatively uncommon injury, most often caused by trauma, but it has also been reported in association with systemic diseases such as diabetes mellitus, rheumatoid arthritis, and local corticosteroid injections [1–4]. Although definitions vary among studies, tendon ruptures are commonly classified according to the interval from injury: acute injuries are generally treated within the first week, subacute injuries between 1 and 3 weeks, and chronic injuries beyond 3 weeks [5, 6]. Primary end‐to‐end repair is generally indicated in acute cases, with satisfactory functional outcomes, including restoration of active hallux extension strength, recovery of range of motion, low complication rates, and return to pre‐injury activity levels [3, 4, 7]. However, in subacute or chronic phases, sustained contraction of the musculotendinous unit, progressive proximal retraction of the tendon stump, scar tissue interposition, and adhesion formation can lead to gap enlargement and loss of tendon elasticity, thereby making primary repair difficult. In such cases, reconstructive procedures such as tendon transfer or graft reconstruction are often required. While acute primary repair has been reported to achieve good‐to‐excellent functional recovery in more than 80%–90% of cases, including restoration of active hallux extension and return to pre‐injury activities, reconstructive procedures in chronic cases are more frequently associated with residual extension lag and reduced strength, with reported full functional recovery rates ranging approximately from 60% to 80% [1, 8, 9].

Anatomical variations of the EHL are common, and the extensor hallucis capsularis (EHC) has been reported in 85%–95% of specimens in previous imaging and cadaveric studies, indicating that it is one of the most common accessory tendons of the EHL [10–13]. The EHC typically runs parallel to the medial side of the EHL, inserting into the capsule of the first metatarsophalangeal (MTP) joint or the base of the proximal phalanx [10, 11, 13]. The functional significance of the EHC remains unclear, but its presence may influence both the mechanism of injury and the reparability of EHL tendon ruptures [8, 10, 11].

Although several studies have reported the optimal timing and techniques for primary repair of EHL tendon injuries, few have addressed how the presence of an accessory tendon such as the EHC influences tendon retraction, surgical feasibility, or postoperative outcomes [2–4, 7]. The EHC, when present, may act as a secondary stabilizer of the hallux extensor mechanism and potentially prevent proximal migration of the ruptured EHL stump. However, its contribution to preserving tendon length or facilitating primary repair after subacute rupture has not been clearly described in the literature. Previous anatomical studies have focused mainly on the morphological classification and insertional variations of the EHC, but clinical reports describing its functional implications in EHL rupture are extremely limited [12, 14]. Therefore, the influence of EHC preservation on surgical decision‐making and prognosis remains uncertain.

Here, we report a rare case of subacute rupture of the EHL tendon in which primary end‐to‐end repair was feasible owing to the presence of a preserved EHC tendon. In this patient, the EHC appeared to have maintained partial continuity of the hallux extensor mechanism, preventing significant retraction of the EHL stumps and allowing tension‐free primary repair even 11 days after injury. The purpose of this case report is to highlight the potential clinical significance of the EHC, specifically the Olewnik Type IIb variant, in EHL tendon injuries and suggest that the presence of this accessory tendon may favorably affect surgical strategy and postoperative recovery.

## 2. Case Presentation

### 2.1. Patient Information and History of Present Injury Mechanism

This case report was produced in accordance with the policy of the local ethical committee of Otaru General Hospital, Otaru, Japan. A 26‐year‐old woman sustained a laceration to the dorsomedial aspect of her left foot after accidentally dropping a kitchen knife. She was treated at a local clinic on the day of injury, where the wound was sutured and no tendon injury was suspected. One week after the injury, she noticed persistent inability to actively extend her left hallux and was referred to our hospital with suspected rupture of the EHL tendon.

### 2.2. Clinical Findings and Imaging Studies

**Figure 1:**
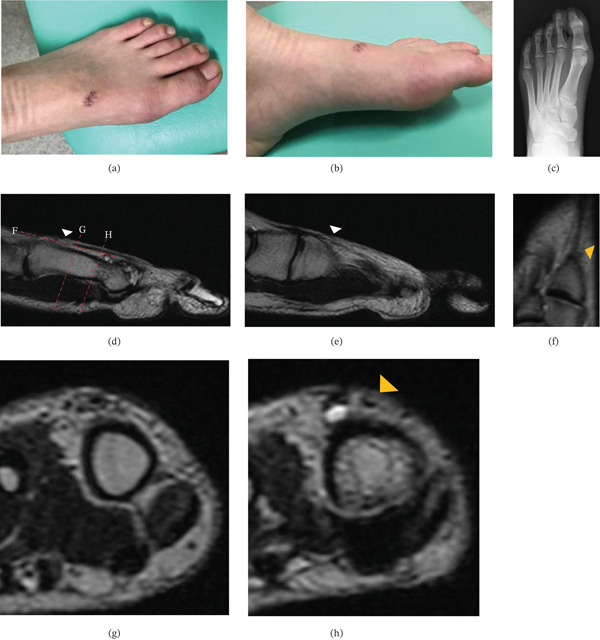
(a, b) Preoperative appearance showing a 1‐cm oblique laceration on the dorsomedial aspect of the midfoot. (c) Plain radiographs showing no osseous abnormalities. (d‐h) T2‐weighted magnetic resonance imaging demonstrating disruption of the extensor hallucis longus (EHL) tendon at the dorsal midfoot (d, e: sagittal; f: coronal; g, h: axial views). In the coronal and axial views, the extensor hallucis capsularis (EHC) is also identified as a thinner medial structure adjacent to the EHL (yellow arrowhead).

Physical examination revealed no swelling, erythema, or tenderness of the left foot. A 1‐cm oblique laceration was observed on the dorsomedial aspect of the midfoot, running from the proximal medial to the distal lateral direction (Figure 1). The proximal stump of the EHL tendon was palpable approximately 5 mm proximal to the wound margin. Active extension of the left hallux was limited, with 20° of extension at the MTP joint and −20° at the interphalangeal (IP) joint, indicating loss of EHL function. Plain radiographs showed no bony abnormalities. Magnetic resonance imaging (MRI) demonstrated disruption of the EHL tendon continuity at the dorsal midfoot. Based on these findings, complete rupture of the EHL tendon was diagnosed.

### 2.3. Surgical Findings and Procedure

**Figure 2:**
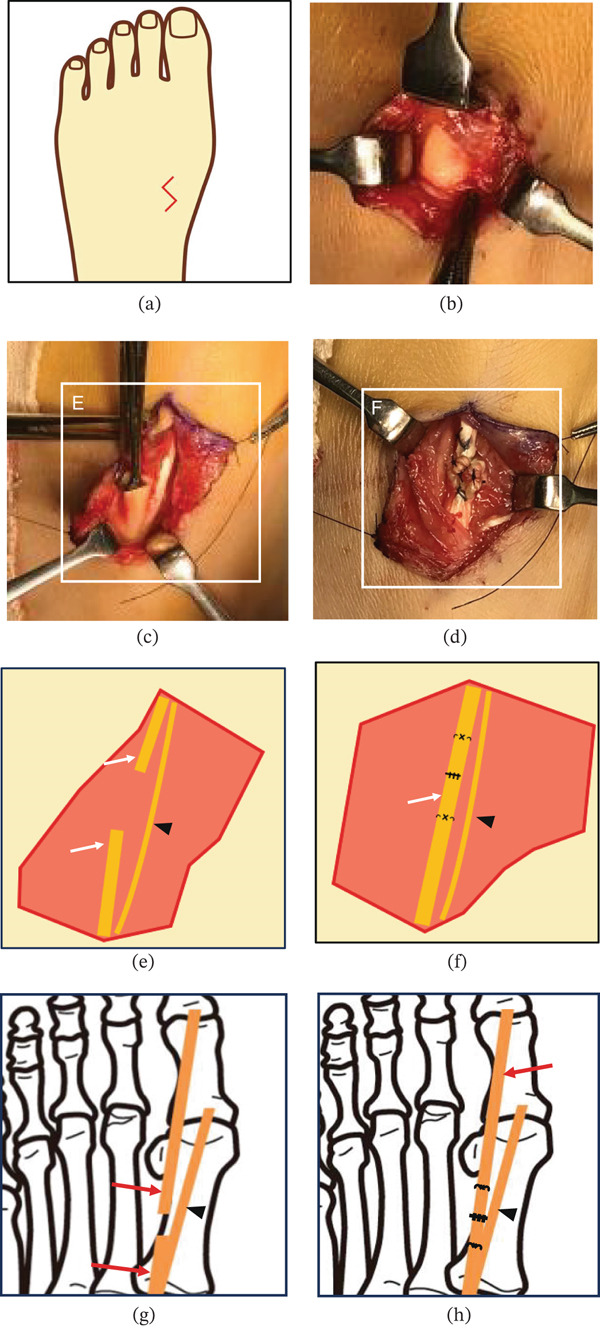
Intraoperative findings. (a) Exposure of the extensor hallucis longus (EHL) tendon stumps through a zigzag incision. (b, c, e) Identification of the extensor hallucis capsularis (EHC) (black arrowheads) running medially to the EHL (white arrows). The thicker central tendon corresponds to the EHL, whereas the thinner medial structure represents the EHC. (d, f) End‐to‐end repair of the EHL performed using a six‐strand Yoshizu Type I technique. (g,h) Schematic illustration of the anatomy of EHL (red arrows) and EHC (black arrowheads). The EHL runs centrally and inserts into the distal phalanx, whereas the EHC courses along the medial side and inserts into the capsule of the first metatarsophalangeal joint or the base of the proximal phalanx.

Under regional anesthesia and pneumatic thigh tourniquet control in the supine position, 1% lidocaine was infiltrated subcutaneously at the wound site. The previous wound was then extended proximally and distally in a zigzag fashion along the course of the EHL tendon (Figure 2). The proximal and distal stumps of the EHL were identified, and the gap was approximately 5 mm with the ankle in a neutral position. On the medial side of the EHL, a thinner accessory tendon running distally toward the MTP joint was observed and identified as the EHC. The EHC inserted distal to the insertion of the extensor hallucis brevis (EHB), and traction on the EHC produced dorsiflexion of the MTP joint, suggesting its attachment to the proximal phalanx. Although dorsiflexion of the ankle reduced the interstump distance, we measured the gap in the neutral position to represent the true interstump distance and to assess reparability under physiologic tension. During the procedure, the ankle was temporarily dorsiflexed to facilitate mobilization and delivery of both tendon ends, thereby reducing the interstump distance before suturing. Given the minimal retraction and intact tendon quality, end‐to‐end tendon repair of the EHL was performed using a six‐strand Yoshizu Type I technique. This method consists of a multistrand core suture configuration in which three longitudinal locking loops are placed on each tendon stump, increasing tensile strength and resistance to gap formation during early mobilization [15]. The repair was completed with the ankle maintained in a neutral position to avoid excessive tension, without the need for tendon lengthening or grafting. The wound was closed in layers, and a short leg splint was applied with the ankle and hallux maintained in a neutral position. Wound disinfection and dressing changes were performed every few days, and sutures were removed at 14 days postoperatively.

### 2.4. Postoperative Course and Outcomes

**Figure 3:**
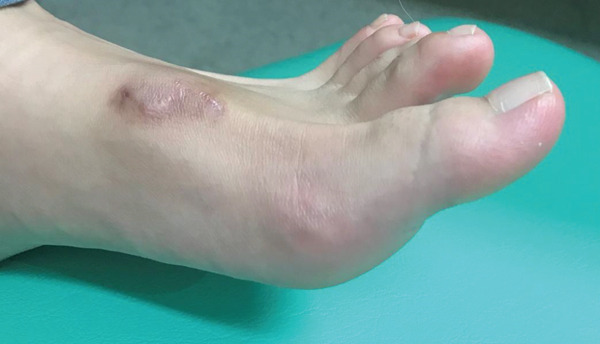
Postoperative view at 12 weeks showing full restoration of hallux dorsiflexion without extension lag.

Postoperatively, the ankle and hallux were immobilized in a neutral position using a short leg splint for 2 weeks, with nonweightbearing ambulation. Two weeks after surgery, the splint was removed, and partial weightbearing and passive hallux extension exercises were initiated. Active flexion of the hallux started at 3 weeks, and full weightbearing was permitted with active hallux extension exercises at 4 weeks postoperatively. At the final follow‐up, 12 weeks after surgery, the patient was able to walk without a limp, and active dorsiflexion of the left hallux reached 50°, comparable to the contralateral side (Figure 3). The American Orthopaedic Foot & Ankle Society (AOFAS) Hallux MTP–IP score improved from 77 preoperatively to 95 postoperatively, and the Japanese Society for Surgery of the Foot (JSSF) Hallux score improved from 82 to 100. No extension lag, stiffness, or rerupture was observed.

## 3. Discussion

This report describes a rare case of subacute EHL tendon rupture in which primary end‐to‐end repair was successfully achieved owing to the presence of a preserved EHC tendon. Although surgery was performed on Day 11 after the injury, the tendon gap remained minimal, and tension‐free repair was possible. The patient demonstrated excellent postoperative recovery, achieving full range of motion and functional restoration without complications.

Olewnik et al. proposed a detailed classification system for EHL accessory tendons based on insertional anatomy [12, 14]. According to Olewnik′s classification, the present case corresponds to Type IIb, in which the medial accessory tendon inserts distal to the EHB insertion. In their cadaveric study, Type II configurations were observed in approximately 70% of specimens, with the Type IIb subtype accounting for roughly 18%–22%. Despite its relatively frequent anatomical occurrence, clinical reports describing EHL rupture in the context of a defined Olewnik subtype are extremely limited. To our knowledge, no previous case report has specifically analyzed the surgical implications of a Type IIb configuration in enabling delayed primary repair. This may suggest that the mechanical contribution of this variant has been underrecognized in surgical decision‐making. Functionally, the EHC contributes to dorsiflexion of the MTP joint but not the IP joint, and in this case likely maintained partial extension tension, preventing proximal retraction of the EHL stump. Therefore, in this case presentation, we achieved a secure repair without creating tension during tendon repair.

The standard treatment for acute EHL rupture is primary end‐to‐end repair when the tendon ends can be approximated without tension; otherwise, tendon transfer or autograft reconstruction is indicated [3, 7, 8]. Poggi and Hall treated acute EHL ruptures with direct end‐to‐end repair using core sutures after adequate mobilization of the tendon stumps and reported restoration of active hallux extension in all patients without rerupture or major complications at follow‐up [3]. Al‐Qattan described primary repair in 17 cases of open laceration, typically employing core suture techniques supplemented by epitendinous sutures [7]. Most patients regained functional hallux extension strength; however, mild extension lag (generally less than 10°) was observed in several cases, although this did not significantly impair daily activities. Wong et al. retrospectively reviewed acute EHL repairs performed with end‐to‐end suture techniques following debridement and tendon mobilization [4]. They reported favorable functional recovery and return to daily activities, although minor residual stiffness and reduced extension range were noted in a subset of patients. In cases where direct approximation was not feasible due to tendon retraction or tissue loss, Nasser et al. described soft‐tissue reconstruction techniques incorporating tendon repair with local tissue rearrangement to address associated defects [2]. Similarly, Kurashige reported reconstruction of chronic EHL rupture using a double‐bundle autograft of the EHC, restoring active extension without rerupture at follow‐up [8]. In contrast, our patient regained full function despite delayed repair, suggesting that preservation of the EHC minimized tendon retraction and allowed a less invasive primary repair approach. The preserved EHC likely acted as a secondary stabilizer, reducing proximal stump migration and facilitating direct primary repair (Figure 2G,H). Additionally, although the Yoshizu technique has been primarily reported in flexor tendon repair, its application to EHL rupture has rarely been described. This high‐strength multistrand configuration may therefore represent a useful option in selected cases requiring increased initial repair strength.

Preoperative imaging is crucial for assessing tendon integrity. MRI remains the standard for evaluating tendon continuity and surrounding soft tissue structures [16, 17]. From a radiological perspective, careful preoperative assessment of tendon gap and stump retraction on MRI may aid in determining reparability. Minimal retraction in subacute cases should raise suspicion of preserved accessory structures such as the EHC, which may influence surgical planning. However, small accessory tendons such as the EHC are often difficult to visualize clearly on MRI due to their small size and variable course. The presence of the EHC may only be inferred indirectly from preserved tendon alignment or minimal proximal retraction despite rupture, rather than being directly visualized. Ultrasonography provides dynamic, real‐time visualization of tendon motion and can serve as a complementary modality when accessory tendons are suspected, although its accuracy depends on operator experience [18, 19]. Although only MRI was used in the present case, combined or selective use of ultrasonography may allow more precise evaluation of tendon stumps and associated anatomical variations.

This report has several limitations. First, it describes a single case, and therefore no definitive conclusions regarding the temporal or mechanical limits of primary repair can be drawn. Second, the precise limits of primary repair in subacute EHL rupture—such as the allowable interval from injury or the maximum interstump gap distance—cannot be determined from this case alone. Third, the influence of accessory tendon morphology was observed in a Type IIb configuration; different types of EHC may not provide the same mechanical effect, and thus the indication for primary repair may vary depending on anatomical variation. Finally, the relatively short follow‐up duration of 12 weeks may not be sufficient to evaluate long‐term functional outcomes, tendon durability, or the risk of late complications such as rerupture or adhesions. Further studies including larger case series and biomechanical analyses are required to clarify these issues.

In conclusion, this case highlights the potential clinical importance of the EHC tendon in managing EHL ruptures. Preservation of the EHC prevented retraction of the EHL stumps and enabled successful primary end‐to‐end repair even on Day 11 after the injury. Surgeons should be aware of this anatomical variation, as its recognition may influence surgical planning and may suggest that primary repair can be considered in selected subacute cases.

**Nomenclature**
EHB: extensor hallucis brevis
EHC: extensor hallucis capsularis
EHL: extensor hallucis longus
IP: interphalangeal
MTP: metatarsophalangeal
MRI: magnetic resonance imaging

## Author Contributions

Ryo Inoue and Yuki Suzuki contributed equally to this study and share first authorship. Ryo Inoue, Yuki Suzuki, and Masanari Hamasaki conceived the original idea. Ryo Inoue, Yuki Suzuki, Masanari Hamasaki, Shigeto Hiratsuka, Takuya Ogawa, and Yukinori Tsukuda performed the clinical assessment. Ryo Inoue, Yuki Suzuki, and Masanari Hamasaki mainly wrote the manuscript, and the study was supervised by Yuki Suzuki, Masanari Hamasaki, Shigeto Hiratsuka, and Yukinori Tsukuda. Katsuro Ura, Masatake Matsuoka, Koji Iwasaki, Tomohiro Onodera, Eiji Kondo, and Norimasa Iwasaki helped supervise the project.

## Funding

No funding was received for this manuscript.

## Disclosure

All the authors made substantial contributions to (1) the conception and design of the study, or acquisition of data, or analysis and interpretation of data; (2) drafting the article or revising it critically for important intellectual content; and (3) final approval of the version to be submitted.

## Ethics Statement

This case report was produced in accordance with the policy of the local ethical committee of Otaru General Hospital, Otaru, Japan. All methods were performed in accordance with the ethical standards as laid down in the Declaration of Helsinki and its later amendments or comparable ethical standards. Written informed consent was obtained from the adult patient for publication of this case report and any accompanying images.

We declare that we have the rights in the work, that we are submitting the work for first publication in the journal, and that it is not being considered for publication elsewhere and has not already been published elsewhere.

## Consent

No written consent has been obtained from the patients as there is no patient identifiable data included in this case report.

## Conflicts of Interest

The authors declare no conflicts of interest.

## Author Biographies

Not applicable.

## Supporting information


**Supporting Information** Additional supporting information can be found online in the Supporting Information section. Reporting guidelines: CARE‐checklist.

## Data Availability

The data that support the findings of this study are available on request due to privacy or ethical restrictions. All data generated or analyzed during this study are included in this published article.
