# Supplementary material for: Primary End‐to‐End Repair of Subacute Extensor Hallucis Longus Tendon Rupture Supported by a Preserved Extensor Hallucis Capsularis: A Case Report
Source: Case Rep Orthop. 2026 Apr 15;2026:4752831. doi: 10.1155/cro/4752831 (PMC13080871; doi:10.1155/cro/4752831)
Supplement: Supplementary file 1 — Supporting Information Additional supporting information can be found online in the Supporting Information section. Reporting guidelines: CARE‐checklist. [file CRO-2026-4752831-s001.pdf]

CARE case report guidelines

CARE Checklist of information to include when writing a case report

(cc) BY-NC-ND

Topic	Item	Checklist item description	Reported on Line
Title	1	The diagnosis or intervention of primary focus followed by the words "case report"	Page 1, Line 1-2
Key Words	2	2 to 5 key words that identify diagnoses or interventions in this case report, including "case report"	Page 5, Line 67-68
Abstract (no references)	3a	Introduction: What is unique about this case and what does it add to the scientific literature?	Page 4, Line 48-54
	3b	Main symptoms and/or important clinical findings	Page 4, Line 54-56
	3c	The main diagnoses, therapeutic interventions, and outcomes	Page 4, Line 56 - Page 5, Line 62
	3d	Conclusion—What is the main "take-away" lesson(s) from this case?	Page 5, Line 62-65
Introduction	4	One or two paragraphs summarizing why this case is unique (**may include references**)	Page 8, Line 109-116
Patient Information	5a	De-identified patient specific information.	Page 8, Line 121 - Page 9, Line 126
	5b	Primary concerns and symptoms of the patient.	Page 8, Line 121 - Page 9, Line 126
	5c	Medical, family, and psycho-social history including relevant genetic information	Page 8, Line 121 - Page 9, Line 126
	5d	Relevant past interventions with outcomes	Page 8, Line 121 - Page 9, Line 126
Clinical Findings	6	Describe significant physical examination (PE) and important clinical findings.	Page 9, Line 129-134
Timeline	7	Historical and current information from this episode of care organized as a timeline	Page 8, Line 121 - Page 9, Line 137
Diagnostic Assessment	8a	Diagnostic testing (such as PE, laboratory testing, imaging, surveys).	Page 9, Line 134-136
	8b	Diagnostic challenges (such as access to testing, financial, or cultural)	N/A
	8c	Diagnosis (including other diagnoses considered)	Page 9, Line 136-137
	8d	Prognosis (such as staging in oncology) where applicable	N/A
Therapeutic Intervention	9a	Types of therapeutic intervention (such as pharmacologic, surgical, preventive, self-care)	Page 9, Line 139 - Page 11, Line 170
	9b	Administration of therapeutic intervention (such as dosage, strength, duration)	Page 9, Line 139 - Page 11, Line 170
	9c	Changes in therapeutic intervention (with rationale)	N/A
Follow-up and Outcomes	10a	Clinician and patient-assessed outcomes (if available)	Page 11, Line 170-175
	10b	Important follow-up diagnostic and other test results	Page 11, Line 170-175
	10c	Intervention adherence and tolerability (How was this assessed?)	N/A
	10d	Adverse and unanticipated events	Page 11, Line 170-175
Discussion	11a	A scientific discussion of the strengths AND limitations associated with this case report	Page 12, Line 178 - Page 15, Line 248
	11b	Discussion of the relevant medical literature **with references**.	Page 12, Line 178 - Page 15, Line 237
	11c	The scientific rationale for any conclusions (including assessment of possible causes)	Page 12, Line 178 - Page 16, Line 254
	11d	The primary "take-away" lessons of this case report (without references) in a one paragraph conclusion	Page 15, Line 249 - Page 16, Line 254
Patient Perspective	12	The patient should share their perspective in one to two paragraphs on the treatment(s) they received	N/A
Informed Consent	13	Did the patient give informed consent? Please provide if requested	**Yes** ☑ **No** ☐
